# Root Functional Specialization in *Dalbergia odorifera* Reveals Localized Molecular Adaptations to Karst Rocky Desertification Stress

**DOI:** 10.3390/plants14243740

**Published:** 2025-12-08

**Authors:** Bizhang Liu, Guili Qin, Yinying He, Wending Tan, Xiaojuan Ming, Shuzhong Yu, Xianyu Yao

**Affiliations:** 1 Guangxi Colleges and Universities Key Laboratory for Cultivation and Utilization of Subtropical Forest Plantation, Guangxi Key Laboratory of Forest Ecology and Conservation, School of Forestry, Guangxi University, Nanning 530004, China; 2409392035@st.gxu.edu.cn (B.L.); 1909302011@st.gxu.edu.cn (G.Q.); 18778228693@163.com (Y.H.); Twd2051172080@163.com (W.T.); mingxiaojuan54@163.com (X.M.); 2 Longli County Forestry Bureau of Qiannan Prefecture, Qiannan 551200, China

**Keywords:** N-fixing tree species, split-root system, limestone rock, macronutrient limitation, root proteomic, metabolomic

## Abstract

*Dalbergia odorifera*, a cornerstone tree species for ecological restoration in karst regions, exhibits remarkable adaptability to karst rocky desertification (KRD) environments characterized by high heterogeneity and nutrient poverty. Yet, the mechanisms underlying its root system’s response to spatially variable KRD stress remain poorly elucidated. In this study, a split-root system was employed to simulate heterogeneous substrate conditions, including loam, uniform gravel (global stress), and partitioned loam/gravel (partial stress). We found that under partial stress, the root system underwent functional specialization, and roots in loam enhanced resource acquisition, whereas roots in gravel significantly elevated stress tolerance. This was supported by increased root:shoot ratio, improved nutrient conservation, and localized upregulation of key enzymes and metabolites. Multi-omics profiling further uncovered profound reprogramming of critical pathways such as phenylpropanoid biosynthesis, fatty acid metabolism, and glutathione metabolism, highlighting robust antioxidant defense and membrane stabilization mechanisms. Our findings demonstrate that *D. odorifera* optimizes resource use in heterogeneous karst habitats through spatial division of labor at the root system level, orchestrated by integrated morphological, physiological, and molecular adaptations. This study provides a novel perspective on plant adaptation to environmental heterogeneity and offers practical insights for cultivating stress-resilient trees and restoring degraded karst ecosystems.

## 1. Introduction

In recent years, the increasing frequency, intensity, and duration of extreme climate events under global climate change have imposed multifaceted stresses on terrestrial ecosystem functioning and plant adaptability [1]. This trend is particularly pronounced in ecologically fragile regions, such as the karst areas of Southwest China. These regions face severe rocky desertification, characterized by highly heterogeneous habitats, shallow soil layers, high bedrock exposure rates, extreme deficiencies in water and nutrients (e.g., phosphorus), and concomitant stresses of high calcium and high pH, creating a highly constrained plant growth environment [2,3,4]. This unique geological context significantly limits the effectiveness of conventional vegetation restoration methods. Consequently, elucidating the systematic adaptation mechanisms of suitable plants under heterogeneous stress has become crucial for enhancing rocky desertification control efficacy and addressing climate change challenges.

Leguminous nitrogen-fixing tree species play a vital role in improving soil structure and nutrient cycling [5]. As a representative species, *Dalbergia odorifera* (*D. odorifera*) not only exhibits strong drought tolerance and calcium endurance but also alleviates nitrogen (N) limitation through rhizobial N fixation [6], making it widely used in ecological restoration projects in karst regions [7]. Numerous field trials have confirmed its significant effectiveness in enhancing soil fertility and facilitating community succession [6,8]. However, current research predominantly focuses on aboveground growth responses and changes in soil physicochemical properties, leaving the systematic regulatory strategies of roots in “soil-rock” heterogeneous substrates, especially the molecular-level response mechanisms, insufficiently explored.

The core strategy of plants coping with heterogeneous environments relies on the plasticity of root functional traits and the coordinated allocation of resources across zones [9]. At the morphological level, roots can adjust biomass allocation, specific root length, and surface area to improve resource capture efficiency [10]. At the physiological level, this involves zonal changes in enzyme activities and the allocation of carbon and nitrogen metabolites. In recent years, the integration of multi-omics technologies has further revealed that the reprogramming of proteins and metabolites constitutes the molecular basis of plant adaptation to complex stresses. For instance, phenylpropanoid biosynthesis and lipid metabolism pathways play key roles in responding to drought and high calcium stress [11], while the regional accumulation of antioxidant metabolites (e.g., flavonoids) helps maintain redox homeostasis [12,13]. Nevertheless, the expression patterns and regulatory networks of these mechanisms in karst-adapted tree species remain largely unknown.

To address these gaps, this study employed *D. odorifera* and utilized a split-root system to simulate the typical “soil-rock fissure” heterogeneous matrix of karst habitats. Four treatments were established, global stress (both compartments with limestone gravel), complete non-stress (both compartments with soil), and partial stress (one compartment with soil, the other with limestone gravel, creating heterogeneity). By integrating root system architecture analysis, physiological enzyme activity assays, elemental stoichiometry, iTRAQ-based quantitative proteomics, and untargeted metabolomics, this research systematically investigates the following scientific questions: (1) How do the biomass allocation and morphological architecture of *D. odorifera* roots respond to heterogeneous stress? Do functional division strategies exist? (2) Do different root zones (stressed vs. non-stressed) exhibit complementary effects in aspects such as nutrient activation (e.g., acid phosphatase activity) and carbon-nitrogen metabolism (e.g., soluble sugars, nitrogen assimilation enzymes)? (3) Which key proteins and metabolites are involved in the regionalized adaptation process? Does the regulatory network involve conserved pathways such as phenylpropanoid synthesis and lipid metabolism? The findings are expected to deepen the understanding of the adaptation mechanisms of woody plants in karst environments and provide a theoretical basis and molecular targets for selecting species and configuring habitat patterns in the ecological restoration of rocky desertification areas. Specifically, we hypothesize that (1) under partial stress, loam roots and gravel roots will adopt distinct stress resistance strategies for adaptation; (2) this adaptation may involve physiological adjustments in nutrient stoichiometry, enzyme activities, and carbon allocation, as well as genomic reprogramming entailing the compartment-specific reconfiguration of proteins and metabolites.

## 2. Results

### 2.1. Changes in Biomass and Root Morphological Characteristics of D. odorifera

As detailed in Table 1, *D. odorifera* seedlings cultivated under Setup A exhibited significantly greater total biomass (dry and fresh mass), along with superior aboveground and belowground biomass, compared to those in Setup B (*p* < 0.05). However, the belowground dry mass and fresh mass in Setup A was statistically comparable to that in Setup C. Notably, Setup B yielded the lowest aboveground biomass (both dry and fresh mass). The root:shoot ratio varied significantly across the setups, with Setup B displaying the highest value, followed by Setup C and then Setup A (Table 1). Evaluation of morphological traits showed a uniform superiority of roots grown in loam substrate across the root characteristics of total length, tip number, and root nodules number (Table 2). At the overall level, treatment 1 was significantly higher than treatment 2. The indicators overall showed that loam was superior to limestone gravel. This hierarchical pattern persisted at the localized treatment level; except for total root surface area and volume, treatment 3 outperformed treatment 4 (in Setup C) in other morphological index assessed, while total root surface area and volume did not differ significantly between treatments 3 and 4.

### 2.2. Changes in Root Mineral Element Content

As shown in Table 3, at the overall level, no significant differences were observed in carbon (C) content among the different treatments. Treatment 1 exhibited significantly higher nitrogen (N) content than treatment 2, while there was no significant difference between treatments 3 and 4. The phosphorus (P) and potassium (K) contents were significantly higher in treatment 1 than other treatments (*p* < 0.05). At the localized treatment level, the contents of P and K were all higher in treatment 3 than in treatment 4, which in treatment 3 were, respectively, higher by 14.52% and 11.40% than treatment 4. With respect to stoichiometric ratios, the C:N ratio in treatment 1 was lower than treatment 2, while treatments 3 and 4 showed no difference at the localized level. The C:P and N:P values were significantly lower in treatment 1 than the other treatments at the overall level. Similarly, at the localized level, the C:P and N:P values were lower in treatment 3 than treatment 4, indicating a consistent trend between overall and localized responses (Table 3).

### 2.3. Changes in Root Activities, Enzyme Activity, Soluble Protein and Carbohydrates Content

**Figure 1:**
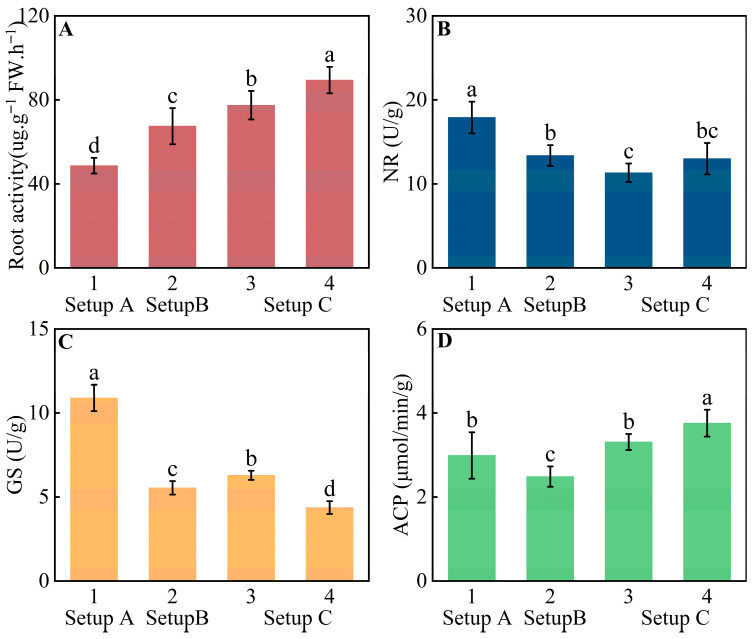
Changes in root activity (**A**), nitrate reductase (**B**), glutamine synthetase (**C**) and acid phosphatase (**D**) in *D. odorifera* roots under treatments 1 to 4. Values are presented as means with standard deviations (*n* = 6). Different letters (a, b, c, d) above the bars indicate the significant differences (ANOVA, SPSS 21.0, *p* < 0.05) between treatments. NR, nitrate reductase; GS, glutamine synthetase; ACP, acid phosphatase.

**Figure 2:**
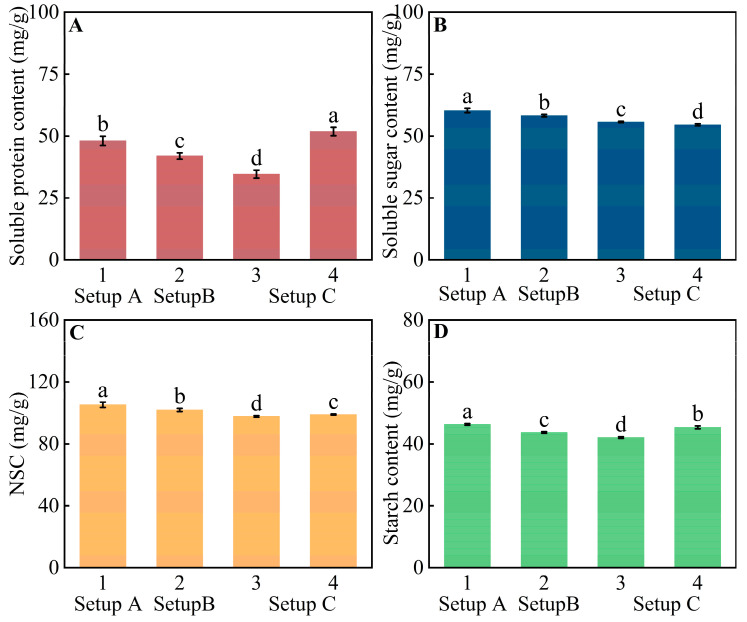
Changes in soluble protein (**A**), soluble sugar (**B**), nonstructural carbohydrates (**C**) and starch (**D**) contents in *D. odorifera* roots under treatments 1 to 4. Values are presented as means with standard deviations (*n* = 6). Different letters (a, b, c, d) above the bars indicate the significant differences (ANOVA, SPSS 21.0, *p* < 0.05) between treatments. NSC, non-structural carbohydrates.

As shown in Figure 1, glutamine synthetase (GS) and acid phosphatase (ACP) activities in the roots of *D. odorifera* were both significantly greater in treatment 1 than treatment 2. Similarly, GS activity was greater in treatment 3 than in treatment 4; but ACP activity was lower in treatment 3 than in treatment 4 at the localized level (*p* < 0.05). Nitrate reductase (NR) activity exhibited a pattern which treatment 1 was significantly greater than treatment 2, while there was no significant difference between treatments 3 and 4 in localized level (*p* < 0.05). The result showed that root activity in both treatments 3 and 4 of Setup C was significantly greater than in Setup A and Setup B. The root activity was significantly lower in Setup 1 than Setup 2 at the overall level, and it exhibited a consistent trend in localized level that treatment 3 was significantly lower than treatment 4. As shown in Figure 2, treatment 1 exhibited significantly higher soluble protein content than treatment 2, but the soluble protein content in treatment 3 was lower than treatment 4 (*p* < 0.05). The soluble sugar content exhibited a consistent trend that treatment 1 was significantly higher than treatment 2 and treatment 3 was significantly higher than treatment 4 (*p* < 0.05). At the overall level, non-structural carbohydrates (NSC) and starch content were significantly higher in treatment 1 than treatment 2, but was significantly lower in treatment 3 than treatment 4 in localized level (*p* < 0.05).

### 2.4. Differential Proteomics Analysis

**Figure 3:**
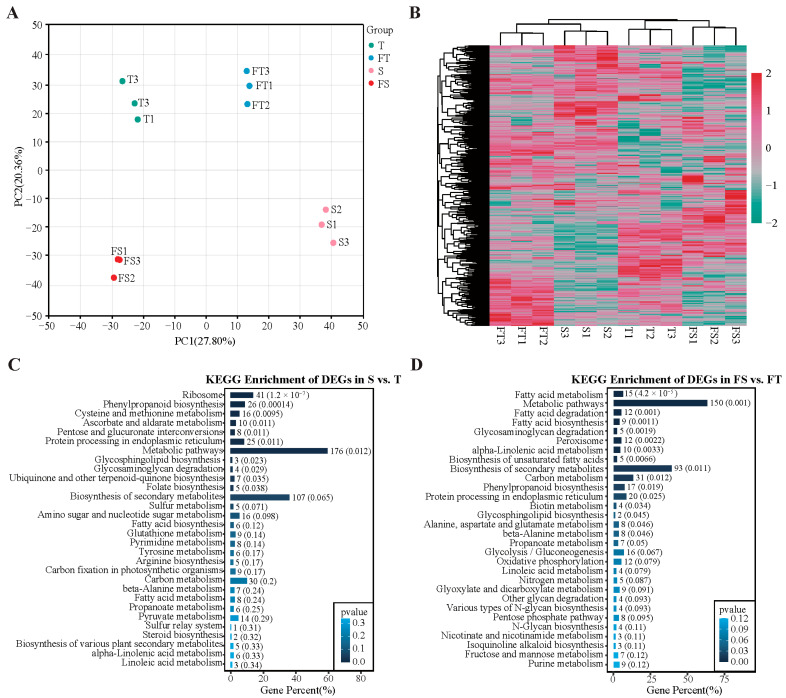
Proteomic analysis of *D. odorifera* roots under different substrate conditions. S: Roots grown entirely in loam substrate. T: Roots grown entirely in limestone gravel substrate. FS: Roots from the loam compartment in the split-root setup. FT: Roots from the limestone gravel compartment in the split-root setup. (**A**,**B**) Principal component analysis (PCA) and hierarchical clustering of differentially expressed proteins, respectively. (**C**,**D**) KEGG pathway enrichment analysis for the S vs. T and FS vs. FT comparisons.

To evaluate the reproducibility and reliability of high-throughput proteomic profiling, quantitative data were assessed using principal component analysis (PCA) and expression clustering analysis (Figure 3A,B). PCA results indicated clear separation between experimental groups and close clustering of replicates within each group, demonstrating high sample reproducibility. Hierarchical clustering of all differentially expressed proteins revealed substantial inter-group variations, with samples from the same group clustering together. Interestingly, the largest expression divergence was observed between the FS and FT groups, suggesting that substrate heterogeneity under the split-root system exerts strong regulatory effects on the root proteome.

Further KEGG pathway enrichment analysis was performed for the comparison groups S vs. T and FS vs. FT (Figure 3C,D). The results showed that differentially expressed proteins in the S vs. T group were primarily involved in ribosomal functions, phenylpropanoid biosynthesis, carbohydrate metabolism, amino acid metabolism, fatty acid metabolism, and secondary metabolite biosynthesis. In the FS vs. FT group, enriched pathways included fatty acid metabolism, peroxisome function, plant secondary metabolism, phenylpropanoid biosynthesis, as well as amino acid and carbohydrate metabolism.

### 2.5. Functional Analysis of Differential Metabolites

**Figure 4:**
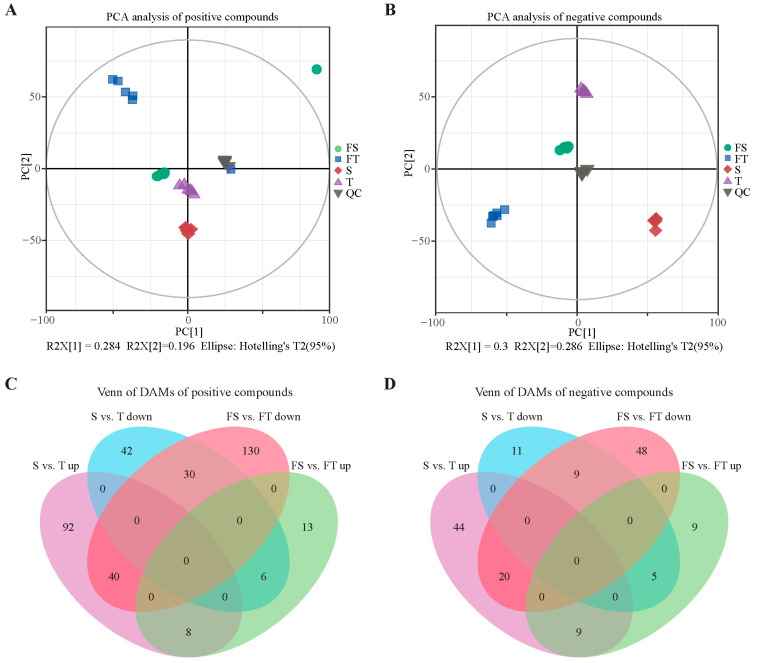
Metabolomic profiling of *D. odorifera* roots under different substrate conditions. S: Roots grown entirely in loam substrate. T: Roots grown entirely in limestone gravel substrate. FS: Roots from the loam compartment in the split-root setup. FT: Roots from the limestone gravel compartment in the split-root setup. QC: Quality control. (**A**,**B**) Principal component analysis (PCA) of metabolic profiles in positive and negative ion modes, respectively. (**C**,**D**) Venn diagrams showing the numbers of commonly up- and down-regulated metabolites in positive and negative ion modes across the S vs. T and FS vs. FT comparisons.

The reproducibility and reliability of the metabolomic data were evaluated through PCA performed on both positive and negative ionization mode datasets (Figure 4A,B). The results showed tight clustering of quality control samples and high intra-group reproducibility, with clear separation between groups despite minor outliers, indicating excellent instrumental stability and data quality. Differentially abundant metabolites were identified and subjected to Venn analysis (Figure 4C,D). In positive ion mode, 40 metabolites were consistently up-regulated and 6 were down-regulated in both the S vs. T and FS vs. FT comparisons. In negative ion mode, 20 metabolites were commonly up-regulated and 5 were down-regulated in both comparison groups.

**Figure 5:**
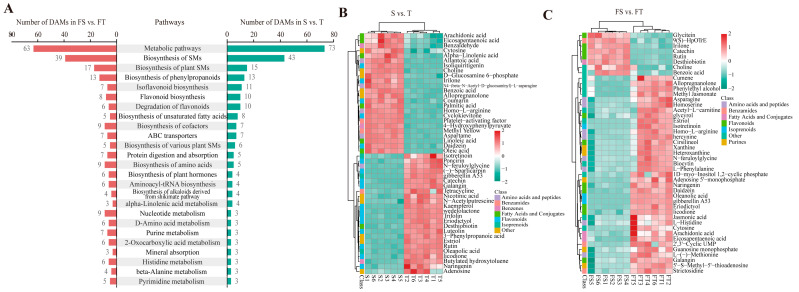
Functional analysis of differential metabolites in *D. odorifera* roots. S: Roots grown entirely in loam substrate. T: Roots grown entirely in limestone gravel substrate. FS: Roots from the loam compartment in the split-root setup. FT: Roots from the limestone gravel compartment in the split-root setup. (**A**) KEGG pathway enrichment analysis for the S vs. T and FS vs. FT comparisons. (**B**,**C**) Abundance clustering heatmaps of differential metabolites identified in the S vs. T and FS vs. FT comparisons.

To further elucidate the functional impact of substrate conditions on root metabolism, KEGG pathway and chemical class enrichment analyses were conducted on the differential metabolites. The results revealed that metabolic pathways affected in both S vs. T and FS vs. FT comparisons were largely consistent, encompassing biosynthesis of secondary metabolites, phenylpropanoid biosynthesis, isoflavonoid and flavonoid synthesis, and unsaturated fatty acid biosynthesis (Figure 5A). Abundance clustering analysis of differential metabolites in the S vs. T comparison indicated that most flavonoids were up-regulated, while the majority of benzenoids and fatty acids/conjugates were down-regulated (Figure 5B). In the FS vs. FT group, up-regulation was observed for most flavonoids, isoprenoids, amino acids/peptides, and purines, whereas fatty acids and conjugates showed mixed regulation patterns (Figure 5C).

**Figure 6:**
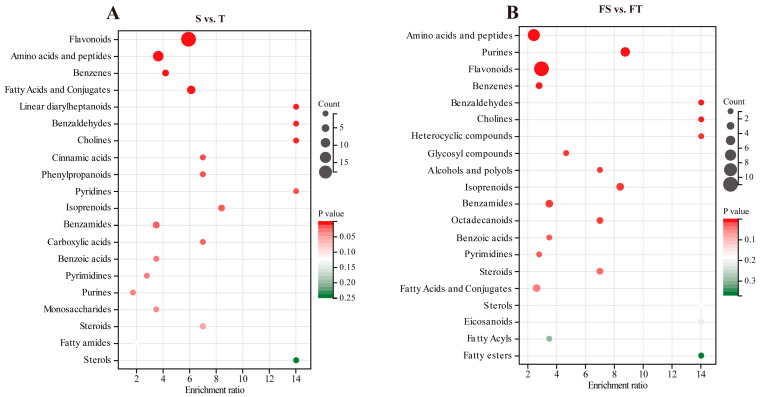
Abundance clustering heatmap of differential metabolites identified in *D. odorifera* roots under different substrate conditions. S: Roots grown entirely in loam substrate. T: Roots grown entirely in limestone gravel substrate. FS: Roots from the loam compartment in the split-root setup. FT: Roots from the limestone gravel compartment in the split-root setup. (**A**,**B**) Abundance clustering heatmap of differential metabolites identified in the global and partial stress comparison (S vs. T and FS vs. FT).

Chemical class enrichment analysis demonstrated that in the S vs. T group, differentially abundant metabolites were most significantly enriched in flavonoids, followed by amino acids and peptides, benzenoids, and fatty acids/conjugates (Figure 6A). In the FS vs. FT group, the most enriched classes were amino acids and peptides, purines, flavonoids, and benzenoids (Figure 6B).

## 3. Discussion

Our study provides a comprehensive analysis of the adaptive strategies employed by *D. odorifera* in response to KRD stress, revealing novel insights into root system functional specialization at morphological, physiological, and molecular levels. The split-root approach enabled unprecedented resolution of spatial responses to heterogeneous substrate conditions, advancing our understanding of plant adaptation mechanisms in karst ecosystems.

### 3.1. Contrasting Root Morphological Strategies in D. odorifera Under Heterogeneous Karst Stress

Traditional models of root plasticity emphasize generalized responses to homogeneous stress conditions [14]. However, by using the root splitting method to handle the triggered local and systemic signal responses [15], we revealed a more complex strategy, functional specialization between root zones under heterogeneous stress. While previous studies in *Zea mays* and *Arabidopsis thaliana* have demonstrated root compensatory growth in heterogeneous nutrient patches [16], our work provides the first evidence in a karst-adapted tree species of simultaneous specialization for both resource acquisition (loam roots) and stress tolerance (gravel roots). This dual strategy represents a significant advance over the optimal partitioning theory, which predicts proportional allocation shifts rather than functional divergence [17]. The maintenance of root biomass under partial stress, despite reduced shoot growth (Table 1), suggests that *D. odorifera* prioritizes root function integrity over overall biomass accumulation, a strategy particularly suited to chronically resource-limited karst environments.

The increased root:shoot ratio in treatment 2 compared to treatment 1 aligns with the global pattern of biomass allocation trade-offs under stress [18]. However, this whole-plant increase in root:shoot ratio is mechanistically decoupled from the compartment-specific morphological specialization we identified. The morphological data from the localized treatments show a clear functional divergence within the same plant (Table 2), roots in the loam substrate (Treatment 3) developed a greater total root length, a higher number of root tips, and more nodules, constituting an acquisition-oriented morphology. In stark contrast, roots in the gravel substrate (Treatment 4) exhibited a reduction in these same traits, a suite of characteristics associated with a conservation or tolerance strategy. This demonstrates that the classic root economics spectrum, which describes trait variation among plants, is also a template for functional specialization within a single individual.

Our findings show that *D. odorifera* does not adopt a uniform root strategy but simultaneously maintains roots with distinct morphological and, consequently, functional roles to optimize resource utilization in a heterogeneous environment. As the organs for conservation and acquisition, the roots in Setup A showed greater root length, surface area, volume, tip number, and root nodule numbers than in Setup B (Table 2). However, in Setup C, only root length, tip number, and nodule number were higher in treatment 3 than in treatment 4, while no significant differences were observed in root surface area and volume. Thus, *D. odorifera* modulates distinct root traits to adapt to heterogeneous environments [17,18].

### 3.2. Physiological Integration of Nutrient Conservation and Metabolic Adjustment

Our results demonstrate a clear physiological dichotomy between root compartments, underpinning their functional specialization. The elemental stoichiometry data provide direct evidence of this divergence. Roots in the gravel substrate (Treatment 4) exhibited significantly lower P and K contents compared to their loam counterparts (Treatment 3), which directly led to their markedly higher C:P and N:P ratios (Table 3). This pattern, the sharp increase in C:P and N:P ratios, confirms a strong nutrient conservation strategy in the gravel roots, aligning with the growth rate hypothesis [19], and reflecting the acute P limitation typical of karst limestone substrates. The elevated C:P ratio suggested a shift towards higher nutrient resorption efficiency and more conservative nutrient use, indicating improved P utilization efficiency under extreme P scarcity [20]. This is particularly critical for P, given its inherent limitation in karst soils. This nutrient-saving mode is further reinforced and operationalized by a profound metabolic reprogramming. The dramatic increase in acid phosphatase activity at the localized level is a direct and efficient response to mobilize scarce P [20,21], thereby enhancing P acquisition and reuse efficiency. The contrasting pattern of nitrate reductase and glutamine synthetase activities slightly increased nitrate reductase but significantly decreased glutamine synthetase (Figure 1), which suggests a disrupted N assimilation pathway [22]. This unique pattern implies that the gravel roots prioritize the initial step of nitrate acquisition but compromise the energy-costly integration of ammonium into amino acids, a metabolic trade-off that may reallocate resources from growth to stress tolerance [23,24].

This metabolic reprogramming is intrinsically linked to a strategic management of carbon resources [25]. The accumulation of starch in gravel-grown roots (Figure 2D) likely provides a stable carbon reserve for sustained maintenance and defense, acting in concert with the observed nutrient conservation strategy. This is robustly evidenced by our metabolomic data, which showed a concerted upregulation of the phenylpropanoid and flavonoid biosynthesis pathways in these roots (Figure 5A), consuming carbon skeletons from primary metabolism to produce protective secondary metabolites. Conversely, the higher content of soluble sugars in loam-grown roots is directly linked to their high root activity and expansive morphology (greater total root length and surface area, Table 2), fuelling the energy-intensive processes of growth and nutrient uptake. This physiological dichotomy is further refined at the metabolic level, while the gravel roots channel carbon into storage and defense compounds like flavonoids and benzenoids (Figure 5B,C), the loam roots likely prioritize the synthesis of amino acids and peptides to support rapid protein synthesis for new tissue development. Thus, the morphological division of labor is precisely mirrored by a systemic physiological division, where carbon is strategically allocated to optimize survival in gravel and growth in loam, enabling *D. odorifera* to master the challenges of heterogeneous karst substrates.

### 3.3. Molecular Mechanisms of Localized Adaptation: From Pathways to Networks

Our integrated proteomic and metabolomic analyses provide a high-resolution view of the molecular reprogramming that dictates the physiological and morphological specialization of the root system. The data reveal not just isolated pathway changes, but a coherent, system-level molecular strategy tailored to the specific challenges of each substrate [26]. Our integrated proteomic and metabolomic analyses reveal a system-level molecular strategy underlying root functional specialization in *D. odorifera* (Figure 3, Figure 4, Figure 5 and Figure 6)*,* which was closely linked to redox homeostasis. Under heterogeneous conditions, the NR activity preserved a comparable level between treatments 3 and 4, despite a decrease in GS activity similar to the uniform gravel setup (Figure 1), indicating a metabolic shift that redirects resources toward defense. Molecularly, this was supported by co-activation of the phenylpropanoid and glutathione metabolism pathways [12,27]. Proteomic data showed upregulation of glutathione-S-transferases and peroxidases, while metabolomics confirmed accumulation of flavonoids and isoflavonoids (Figure 3, Figure 4, Figure 5 and Figure 6). Thus, root specialization relies on a coordinated system wherein altered enzyme activities bolster antioxidant synthesis and regeneration [28], maintaining redox balance and cellular integrity under stress. In the gravel roots, a cornerstone of this molecular adaptation is the robust co-activation of the phenylpropanoid pathway at both the protein and metabolite levels [27,29]. Our KEGG enrichment analysis specifically highlighted phenylpropanoid biosynthesis as a key pathway differentiating the FS and FT groups (Figure 3D). This proteomic signature is directly validated by the metabolomic data, which showed a distinct up-regulation of flavonoids and isoflavonoids in the corresponding comparison (Figure 5A,C). This coherent multi-omics evidence confirms a targeted investment in secondary metabolism [12]. The resultant production of flavonoid and isoflavonoid antioxidants provides a crucial chemical shield against oxidative stress, a common consequence of abiotic adversity [12,30]. Concurrently, the upregulation of biosynthetic enzymes for lignin, a key end-product of the phenylpropanoid pathway, points to a complementary strategy of physical cell wall reinforcement [27,31,32]. This dual chemical-physical defense paradigm is precisely tailored to endure the mechanical and oxidative challenges inherent to the gravel substrate.

Our proteomic data showed a significant enrichment of fatty acid metabolism pathways (Figure 3C,D), a finding that is functionally explained by the metabolomic observation of accumulated unsaturated fatty acids (includingα-linolenic, linoleic, and oleic acid) in the gravel roots [33]. This coordinated shift serves a critical dual purpose. First, the incorporation of these unsaturated fatty acids into membrane lipids helps maintain membrane fluidity and function under stress-induced rigidity [33,34]. Second, these same fatty acids act as precursors for the synthesis of jasmonate phytohormones, which orchestrate defense responses, and for the synthesis of cutin and suberin polymers that form protective barriers at the root–soil interface [35,36]. This molecular evidence directly supports the observed reduction in specific root length and increased tissue density, as suberization and lignification are key drivers of these morphological traits [37,38]. Our KEGG enrichment analysis of the FS vs FT comparison (Figure 3D) specifically highlighted glutathione metabolism as a core responsive pathway, demonstrating how the molecular response in gravel-grown roots is fundamentally coordinated with their physiological role [39,40]. The significant upregulation of glutathione-S-transferases and glutathione peroxidases, as revealed by our proteomic data, provides the essential enzymatic machinery for detoxifying reactive oxygen species generated under karst stress [39]. Crucially, our multi-omics approach revealed that this antioxidant defense is not standalone but is deeply integrated with primary metabolism through the parallel upregulation of cysteine and methionine biosynthetic pathways [40,41]. This metabolic integration, evident at the proteomic level, ensures a sustained supply of precursors for glutathione synthesis, creating a continuous antioxidant cycle [39,41]. This system performs the critical function of protecting other key adaptation mechanisms identified in our study, it safeguards the integrity of the unsaturated fatty acids that maintain membrane fluidity and preserves the activity of enzymes driving the phenylpropanoid biosynthesis pathway for lignin and flavonoid production. Thus, the glutathione system emerges as a foundational component of the root’s adaptive network; without its protective capacity, the benefits of lipid remodeling for membrane stability and phenylpropanoid deposition for structural reinforcement would be compromised by oxidative damage, ultimately undermining the physiological specialization observed between root compartments [42].

In conclusion, the molecular landscape we have delineated is not a collection of independent events but a highly coordinated network [43]. The simultaneous enhancement of phenylpropanoid, lipid, and glutathione metabolism in the gravel roots constitutes a synergistic defense triad, the phenylpropanoid pathway builds physical and chemical barriers, lipid metabolism ensures membrane stability and signaling, and the glutathione system provides the essential oxidative damage control. This integrated molecular network provides a mechanistic explanation for the remarkable stress tolerance of the gravel-specialized roots, offering a deep molecular rationale for the functional specialization observed at the whole-plant level.

### 3.4. Ecological Implications and Future Perspectives

The sophisticated adaptive strategy exhibited by *D. odorifera* provides important insights for ecological restoration in karst regions. The species’ ability to maintain root function through functional specialization rather than mere stress tolerance represents a valuable trait for revegetation efforts. The molecular mechanisms identified offer potential targets for genetic improvement programs aimed at enhancing stress resilience in other species.

However, several questions warrant further investigation. Future research should examine the temporal dynamics of the observed adaptations, particularly whether the functional specialization is maintained during prolonged stress exposure. The role of root-to-root signaling in coordinating the responses between compartments remains unexplored and represents an exciting avenue for future research. Field studies in natural karst environments would help validate these controlled experimental findings and assess their ecological relevance under realistic conditions. Additionally, exploring the interactions between *D. odorifera*’s root adaptations and rhizosphere microbial communities could provide valuable insights for developing microbial-assisted restoration strategies. The potential trade-offs between stress tolerance and growth under optimal conditions also merit investigation, as these relationships will influence the species’ performance in restoration scenarios.

## 4. Materials and Methods

### 4.1. Plant Materials and Experimental Design

The experiment was conducted at the experimental base of Guangxi University (108°22′ E, 22°48′ N). One-year-old *D. odorifera* seedlings of uniform growth vigor (height: 42 ± 0.7 cm; ground diameter: 3.6 ± 0.9 mm) from a common provenance (Nanning Arboretum, Nanning, China) were selected. All primary roots were removed prior to transplantation. Seedlings were transplanted into opaque plexiglass root boxes (30 cm × 15 cm × 40 cm, L × W ×H). A split-root system was implemented by physically separating the two compartments within each root box using a plexiglass divider to prevent the exchange of nutrients, water, and other substances between the loam and gravel substrates.

**Figure 7:**
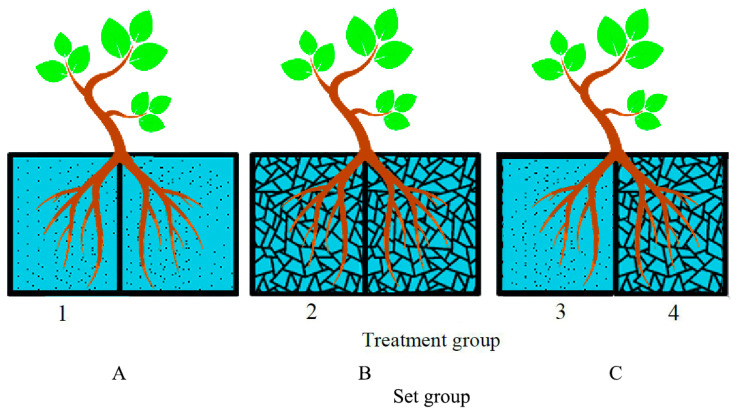
Schematic diagram of split root culture system. (**A**) Both compartments filled with loam; (**B**) Both compartments filled with limestone gravel; (**C**) Left compartment with loam and right compartment with limestone gravel. Note: Treatments 1 and 3 are loam matrix; Treatments 2 and 4 are gravel matrix.

The split-root system (Figure 7) comprised two substrate types: loam and limestone gravel (simulating an extreme rocky desertification environment without soil in karst fissures). Three experimental setups (A, B, C) with four treatments (1 to 4) were established as follows: Setup A: Both compartments filled with loam (non-stressed control); Setup B: Both compartments filled with limestone gravel (global stress); Setup C: Left compartment with loam (Treatment 3, non-stressed zone) and right compartment with limestone gravel (Treatment 4, stressed zone), representing partial stress.

Given that it was conducted in a greenhouse, each root box contained one seedling, with 20 replicates per setup (60 seedlings in total). Planting was conducted in March 2021. The root system of each seedling was evenly divided into two parts based on root number [44]. The loam (dark brown limestone soil) and limestone gravel (particle size ≈ 0.5 cm) were collected from Dalucun Village, Wucun Town, Tianyang District, Baise City (23°33.578′ N, 106°50.315′ E), and a limestone quarry in Mashan County (107°41′–108°29′ E, 23°24′–24°2′ N), both located in rocky desertification-prone regions of Guangxi Zhuang Autonomous Region, China. The physicochemical properties of the two substrates are summarized in Table 4.

To ensure seedling survival and normal growth under local climatic conditions, a sprinkler irrigation system was implemented. During the initial establishment phase, irrigation was conducted for 6 min every 2 h. In the subsequent growth period, irrigation was adjusted to three sessions per day (morning, noon, and evening), each lasting 15 min. Uniform field management practices were maintained throughout the experimental period, including regular removal of weeds. No fertilization was applied. All root boxes were arranged in a completely randomized design and periodically rotated to minimize the impact of environmental heterogeneity.

### 4.2. Biomass and Composition Analysis

After 120 days of growth adaptation, a total of 18 representative *D. odorifera* seedlings (six from each substrate setup) exhibiting vigorous growth and absence of pests or diseases were selected. Roots, stems, and leaves were carefully separated, washed, and gently blotted to remove surface moisture. Fresh weights of each tissue were measured using an electronic balance. Root samples were scanned with a root scanner (Epson Perfection V700 Photo, Epson, Suwa, Nagano, Japan) to obtain morphological parameters. All samples were then placed in a constant-temperature oven, deactivated at 105 °C for 30 min, and subsequently dried at 75 °C to a constant weight for dry weight measurement. The average values were calculated, and the root:shoot ratio was determined.

The mineral element content in the roots was measured according to the methods described by Zhou [45] and Li et al. [46]. Organic carbon content was determined using the ferrous sulfate titration method. Nitrogen, phosphorus, and potassium were determined using the Kjeldahl method, the molybdenum antimony colorimetric method, and flame atomic absorption spectrometry, respectively [47]. Root enzyme activities, including nitrate reductase (NR), acid phosphatase (ACP), and glutamine synthetase (GS), were quantified using commercial assay kits (Bonoheng Biotech Co., Ltd, Nanjing, China; Cat# YX-C-A800, YX-C-B001, YX-C-A807) according to the manufacturer’s instructions. Root activity was evaluated by measuring the oxidation of α-naphthylamine (α-NA) [48]. Root segments were incubated in an α-NA solution, and the reaction mixture was sampled at 0 and 60 min. The α-NA concentration in the samples was quantified colorimetrically at 540 nm after a diazo-coupling reaction with sulfanilic acid and sodium nitrite. Root activity was determined from the decrease in α-NA concentration over time and expressed as µg α-NA oxidized g^−1^FW h^−1^. Starch and soluble sugar contents were measured using acid hydrolysis-colorimetry and anthrone-sulfuric acid methods, respectively, following the protocols outlined in Guidance of Plant Physiology Experiments [49]. The total non-structural carbohydrate (NSC) content was calculated as the sum of soluble sugars and starch.

### 4.3. Proteomics Analysis

#### 4.3.1. Root Sample Protein Pretreatment

Root samples of *D. odorifera* were thoroughly ground into powder in liquid nitrogen. An appropriate volume of lysis buffer (8 M urea, 2 mM EDTA, 10 mM DTT, 1% protease inhibitor) was added, followed by ultrasonic disruption on ice for 1–2 min. After incubation for 30 min, the homogenate was centrifuged at 12,000 rpm and 4 °C for 10 min. The supernatant was collected as the crude protein extract. Protein concentration was determined using a BCA assay kit (Beyotime Biotechnology, Shanghai, China) according to the manufacturer’s instructions. For each sample, 100 μg of protein was precipitated with 3–5 volumes of prechilled acetone at –20 °C for 3 h. After centrifugation at 12,000 rpm and 4 °C for 10 min, the pellet was air-dried briefly and redissolved in 30 μL of dissolution buffer (8 M urea, 100 mM TEAB). Disulfide bonds were reduced with 10 mM DTT at 37 °C for 60 min, followed by alkylation of free thiols with 25 mM iodoacetamide (IAM) in the dark at room temperature for 30 min. The urea concentration was diluted to below 2 M using 10 mM TEAB. Trypsin was added in two steps (enzyme-to-protein ratio of 1:50 overnight and 1:100 for 6 h) at 37 °C for complete digestion. Peptides were acidified with hydrochloric acid, desalted using a C18 SPE column (Welch Materials, Shanghai, China), and dried under vacuum. The peptides were labeled using an iTRAQ kit (Beyotime Biotechnology, Shanghai, China) according to the manufacturer’s instructions and then pooled. Labeled peptides were prefractionated into 10 fractions via HPLC using a Waters XBridge Shield C18 RP column (Bridge Peptide BEH C18, 130Å, 3.5 µm, 4.6 × 250 mm; Waters Corporation, Milford, MA, USA) with a 90-min mobile phase gradient. The separated peptides were dried again under vacuum.

#### 4.3.2. LC-MS/MS Peptide Analysis

Each fraction was redissolved in mobile phase A, centrifuged at 12,000 rpm for 2 min, and the supernatant was subjected to LC-MS/MS analysis. An Ultimate RSLCnano 3000 ultra-high-performance liquid chromatography system (Thermo Fisher Scientific, Waltham, MA, USA) coupled to a Q Exactive HF mass spectrometer (Thermo Fisher Scientific, Bremen, Germany) was used. Approximately 2 μg of peptides was loaded onto a Thermo Acclaim PepMap 100 C18 column (2 µm, 75 µm × 20 mm; Thermo Fisher Scientific, Sunnyvale, CA, USA) for enrichment with mobile phase A (100% H_2_O, 0.1% formic acid). Separation was performed on a Thermo Acclaim PepMap RSLC C18 column (2 µm, 75 µm × 20 mm; Thermo Fisher Scientific, Sunnyvale, CA, USA) with a gradient of mobile phase B (80% acetonitrile, 0.1% formic acid) over 78 min at a flow rate of 300 nL/min and a column temperature of 40 °C. Peptides were ionized using an ESI ion source at 2 kV, filtered through an ion transfer system, and selected by a quadrupole before being analyzed in an Orbitrap mass analyzer(Thermo Fisher Scientific, Bremen, Germany). Full MS/dd-MS^2^ mode was applied with a full scan range of 350–2000 *m*/*z* at a resolution of 60,000; AGC target was set to 10^6^. The top 20 precursor ions were selected for fragmentation with NCE 30, and fragment ions were scanned from 0 to 2000 *m*/*z* at a resolution of 15,000; AGC target was 10^5^. Charge states between 2 and 6 were included, and dynamic exclusion was set to 20 s.

#### 4.3.3. Protein Identification and Data Analysis

During the mass spectrometry analysis phase, due to budget constraints, we performed sample pooling for the six biological replicates in each treatment group: two randomly selected samples were pooled in equal amounts, ultimately yielding three pooled samples per treatment group, resulting in a total of twelve samples for protein identification. Spectra were matched against the *D. odorifera* protein database using Sequest within Proteome Discoverer 2.0. Trypsin/P was specified as the enzyme with up to two missed cleavages allowed. Peptide FDR was set to 0.01. Mass tolerances were 20 ppm for precursor ions and 0.02 Da for fragment ions. Fixed modification included carbamidomethylation of cysteine; variable modifications included oxidation of methionine and N-terminal acetylation. Differentially expressed proteins were identified with thresholds of |Fold Change| > 1.5 and *p* < 0.05. KEGG pathway annotation was performed using the KAAS online service tool (available at https://www.genome.jp/tools/kaas/, accessed on 19 March 2025). Enrichment analysis of KEGG pathways was conducted using a two-tailed Fisher’s exact test against all identified proteins.

### 4.4. Metabolomics Analysis

#### 4.4.1. Metabolite Extraction from Root Samples

Root samples were ground in liquid N, and 1 mL of 50% methanol extraction solvent was added. The mixture was vortexed for 5 min and sonicated for 15 min. After centrifugation at 12,000 rpm and 4 °C for 10 min, the supernatant was incubated at −20 °C for 30 min and centrifuged again under the same conditions. The final supernatant was transferred to an injection vial for LC-MS/MS analysis.

#### 4.4.2. LC-MS/MS Analysis

Metabolites were separated using an Ultimate 3000 UPLC system (Thermo Fisher Scientific, Waltham, MA, USA) coupled to a Q Exactive mass spectrometer (Thermo Fisher Scientific, Bremen, Germany). Separation was performed on a Waters ACQUITY UPLC HSS T3 C18 column (1.8 µm, 2.1 mm × 100 mm; Waters Corporation, Milford, MA, USA) with a 20-min gradient of mobile phase B (100% methanol, 0.1% formic acid) at a flow rate of 300 μL/min and 40 °C. Both positive and negative ion modes were applied in full MS/dd-MS^2^ mode. HESI ionization was used with spray voltages of 2.0 kV (positive) and 1.5 kV (negative); ion transfer temperatures were 320 °C and 250 °C, respectively. Full scan ranged from 100 to 1000 *m*/*z* at 70,000 resolution; AGC target was 3 × 10^6^. The top 5 precursors were fragmented at NCE 40, 60, and 80; MS^2^ resolution was 17,500 with an AGC target of 10^5^. Dynamic exclusion was set to 10 s.

#### 4.4.3. Metabolite Identification and Data Analysis

Raw data were processed using Compound Discoverer 3.0. Metabolites were identified by matching against the MzCloud and ChemSpider (including BioCyc, DrugBank, KEGG, and PlantCyc) databases with a mass tolerance of 5 ppm. Multivariate statistical analysis (PCA and PLS-DA) and quality control were performed in SIMCA. Differentially abundant metabolites were selected with VIP > 1, |Fold Change| > 2, and *p <* 0.05. Functional annotation and pathway enrichment analysis were conducted using Metabo Analyst (version 6.0, https://www.metaboanalyst.ca, accessed on 5 May 2025). A two-tailed Fisher’s exact test was used to evaluate the enrichment of metabolite classes and KEGG pathways. 

### 4.5. Statistical Analysis

Data were collated using Microsoft Excel 2010. Statistical analysis was performed with SPSS 21.0 (IBM Corp., Armonk, NY, USA). One-way ANOVA followed by multiple comparisons (*p* < 0.05) was applied using the Least Significant Difference (LSD) tests. Graphs were generated using Origin (Version 8.5, OriginLab Corp., Northampton, MA, USA).

## 5. Conclusions

This study demonstrates that *D. odorifera* employs a sophisticated multi-level strategy to thrive in heterogeneous karst environments. Through functional specialization of its root system, the species simultaneously enhances resource acquisition in favorable microsites and stress tolerance in impoverished substrates. Key adaptations include increased root:shoot ratio, improved nutrient use efficiency, and compartment-specific regulation of enzymatic activities and carbon allocation. Integrated proteomic and metabolomic analyses reveal that these responses are underpinned by coordinated molecular reprogramming, involving phenylpropanoid biosynthesis for antioxidant defense and cell wall reinforcement, lipid metabolism for membrane stability, and glutathione pathways for oxidative stress detoxification. These findings provide mechanistic insights into the remarkable resilience of karst-adapted species and offer valuable implications for ecological restoration strategies in degraded landscapes. The study highlights the importance of considering spatial heterogeneity in plant stress responses and suggests that functional root specialization represents a key adaptation for survival in environmentally challenging ecosystems.

## Figures and Tables

**Table 1:** Biomass allocation between underground (root) and aboveground tissues of *D. odorifera* seedlings under different treatments. The numbers in the table represent the mean ± standard deviation (*n* = 6) of each variable. Different lowercase letters within the same row indicate significant differences (ANOVA, SPSS 21.0, *p* < 0.05) among treatments.

Indicator		Setup A	Setup B	Setup C
Total Dry Mass (g)		20.3 ± 3.2 a	8.1 ± 1.0 c	14.5 ± 3.9 b
Total Fresh Mass (g)		57.4 ± 7.2 a	20.4 ± 2.1 c	42.2 ± 11.3 b
Above ground	Dry Mass (g)	16.5 ± 2.9 a	5.5 ± 0.7 c	11.1 ± 3.0 b
	Fresh Mass (g)	44.8 ± 6.6 a	12.2 ± 1.5 c	29.2 ± 7.8 b
Below ground	Dry Mass (g)	3.8 ± 0.4 a	2.6 ± 0.4 b	3.4 ± 0.9 ab
	Fresh Mass (g)	12.6 ± 0.9 a	8.2 ± 0.9 b	13.0 ± 3.5 a
Root:Shoot ratio		0.2 ± 0.03 c	0.5 ± 0.07 a	0.3 ± 0.01 b

**Table 3:** Root nutrient content and stoichiometric ratios of *D. odorifera* seedlings under different treatments. The numbers in the table represent the mean ± standard deviation (*n* = 6) of each variable. Different lowercase letters within the same row indicate significant differences (ANOVA, SPSS 21.0, *p* < 0.05) among treatments.

Element Content	Setup A	Setup B	Setup C	
	Treatment 1	Treatment 2	Treatment 3	Treatment 4
C (g kg^-1^)	452.7 ± 8.8 a	450.3 ± 2.0 a	450.7 ± 3.3 a	455.0 ± 5.8 a
N (g kg^-1^)	14.9 ± 0.3 a	13.0 ± 1.6 b	12.4 ± 0.6 bc	11.9 ± 0.7 c
P (g kg^-1^)	2.1 ± 0.07 a	1.4 ± 0.24 bc	1.5 ± 0.02 b	1.3 ± 0.04 c
K (g kg^-1^)	7.1 ± 0.12 a	6.3 ± 0.62 b	5.6 ± 0.02 c	5.0 ± 0.10 d
C:N	30.5 ± 0.6 c	35.0 ± 4.4 b	36.5 ± 1.8 ab	38.5 ± 2.5 a
C:P	217.6 ± 5.4 c	321.0 ± 49.7 ab	297.2 ± 3.1 b	343.8 ± 7.8 a
N:P	7.1 ± 0.3 c	9.2 ± 0.7 a	8.2 ± 0.5 b	9.0 ± 0.8 a

**Table 2:** Root morphology of *D. odorifera* seedlings under different treatments. The numbers in the table represent the mean ± standard deviation (*n* = 6) of each variable. Different lowercase letters within the same row indicate significant differences (ANOVA, SPSS 21.0, *p* < 0.05) among treatments. Note: For the unification of data standards, the data of treatments 1 and 2 in the whole root system will be halved.

Indicator	Setup A	Setup B	Setup C	
	Treatment 1	Treatment 2	Treatment 3	Treatment 4
Total root length (cm)	1492.3 ± 211.0 a	597.9 ± 44.5 c	898.0 ± 137.3 b	637.5 ± 200.6 c
Total root surface area (cm^2^)	268.0 ± 36.6 a	165.9 ± 31.2 b	256.6 ± 37.9 a	208.5 ± 78.7 ab
Root volume (cm^3^)	4.0 ± 0.5 b	3.0 ± 0.3 c	6.1 ± 1.1 a	5.6 ± 2.4 abc
Root tip number	4303.5 ± 472.8 a	966.8 ± 125.8 c	1349.3 ± 294.5 b	844.8 ± 197.7 c
Number of root nodules	35.8 ± 5.8 b	28.0 ± 2.5 c	51.0 ± 8.4 a	10.2 ± 1.0 d

**Table 4:** Physicochemical properties of two substrates.

Growth Media	Soil	Gravel
pH	7.02	8.21
Total N (g kg^−1^)	1.33	Extremely low
Total P (g kg^−1^)	0.38	0.002
Total K (g kg^−1^)	28.4	0.105
Organic matter (g kg^−1^)	23.87	/
Field capacity (%)	29.0	0.34
Bulk density (g cm^−3^)	1.27	1.14
Soil density (10 cm Kpa^−1^)	187.78	575.22
Total soil porosity (%)	51.91	57.06
Soil aeration pores (%)	25.34	56.64
Soil water-holding pores (%)	26.57	0.42

## Data Availability

The data generated or analyzed in this study are included in this article. Other materials that support the findings of this study are available from the corresponding author on reasonable request.

## Abbreviations

The following abbreviations are used in this manuscript:

KRD	Karst Rocky Desertification
NR	Nitrate Reductase
GS	Glutamine Synthetase
ACP	Acid Phosphatase
NSC	Non-Structural Carbohydrate
GST	Glutathione S-Transferase
ROS	Reactive Oxygen Species
S	Roots grown entirely in loam substrate
T	Roots grown entirely in limestone gravel substrate
FS	Roots from the loam compartment in the split-root setup
FT	Roots from the limestone gravel compartment in the split-root setup

## References

[1] Qu Q., Xu H., Ai Z., Wang M., Wang G., Liu G., Geissen V., Ritsema C.J., Xue S. (2023). Impacts of extreme weather events on terrestrial carbon and nitrogen cycling: A global meta-analysis. Environ. Pollut..

[2] Xie L.W., Zhong J., Chen F.F., Cao F.X., Li J.J., Wu L.C. (2015). Evaluation of soil fertility in the succession of karst rocky desertification using principal component analysis. Solid Earth.

[3] Qi D., Wieneke X., Tao J., Zhou X., Desilva U. (2018). Soil pH Is the Primary Factor Correlating With Soil Microbiome in Karst Rocky Desertification Regions in the Wushan County, Chongqing, China. Front. Microbiol..

[4] Li D., Li X., Du X., Zhang X., Wang J., Dungait J.A.J., Quine T.A., Green S.M., Wen X., Yang Y. (2023). Response of heterotrophic respiration to vegetation restoration in a karst area of SW China. Land Degrad. Dev..

[5] Cui Y., Yan Y., Wang S., Zhang H., He Y., Jiang C., Fan R., Ye S. (2023). Mixed Eucalyptus Plantations in Subtropical China Enhance Phosphorus Accumulation and Transformation in Soil Aggregates. Front. For. Glob. Change.

[6] Yao X., Li Y., Liao L., Sun G., Wang H., Ye S. (2019). Enhancement of nutrient absorption and interspecific nitrogen transfer in a *Eucalyptus urophylla* × *eucalyptus grandis* and *Dalbergia odorifera* mixed plantation. For. Ecol. Manag..

[7] Yu S., Yu K., Yang Z. (2022). Physiological Characteristics and Proteome of *Dalbergia odorifera* in Loam and Gravel Substrates. Horticulturae.

[8] Li M., You Y., Tan X., Wen Y., Yu S., Xiao N., Shen W., Huang X. (2022). Mixture of N2-fixing tree species promotes organic phosphorus accumulation and transformation in topsoil aggregates in a degraded karst region of subtropical China. Geoderma.

[9] Colombi T., Pandey B.K., Chawade A., Bennett M.J., Mooney S.J., Keller T. (2024). Root plasticity versus elasticity—When are responses acclimative?. Trends Plant Sci..

[10] Freschet G.T., Bellingham P.J., Lyver P.O.B., Bonner K.I., Wardle D.A. (2013). Plasticity in above- and belowground resource acquisition traits in response to single and multiple environmental factors in three tree species. Ecol. Evol..

[11] Sharma A., Shahzad B., Rehman A., Bhardwaj R., Landi M., Zheng B. (2019). Response of Phenylpropanoid Pathway and the Role of Polyphenols in Plants under Abiotic Stress. Molecules.

[12] Agati G., Azzarello E., Pollastri S., Tattini M. (2012). Flavonoids as antioxidants in plants: Location and functional significance. Plant Sci..

[13] Rao M.J., Duan M., Zhou C., Jiao J., Cheng P., Yang L., Wei W., Shen Q., Ji P., Yang Y. (2025). Antioxidant Defense System in Plants: Reactive Oxygen Species Production, Signaling, and Scavenging During Abiotic Stress-Induced Oxidative Damage. Horticulturae.

[14] López-Bucio J.S., Ravelo-Ortega G., López-Bucio J. (2022). Chromium in plant growth and development: Toxicity, tolerance and hormesis. Environ. Pollut..

[15] Kafle A., Frank H.E.R., Rose B.D., Garcia K., Gifford M. (2022). Split down the middle: Studying arbuscular mycorrhizal and ectomycorrhizal symbioses using split-root assays. J. Exp. Bot..

[16] Giehl R.F.H., von Wiren N. (2014). Root Nutrient Foraging. Plant Physiol..

[17] BLOOM A., CHAPIN F., MOONEY H. (1985). Resource Limitation in Plants—An Economic Analogy. Annu. Rev. Ecol. Syst..

[18] Poorter H., Niklas K.J., Reich P.B., Oleksyn J., Poot P., Mommer L. (2012). Biomass Allocation to Leaves, Stems and Roots: Meta-Analyses of Interspecific Variation and Environmental Control. New Phytol..

[19] Elser J., O’Brien W., Dobberfuhl D., Dowling T. (2000). The Evolution of Ecosystem Processes: Growth Rate and Elemental Stoichiometry of A Key Herbivore in Temperate and Arctic Habitats. J. Evol. Biol..

[20] Yuan L., Xi-huan L., Xing S., Cai-ying Z. (2012). Variation of Acid Phosphatase Activity and Analysis of Genotypic Difference in P Efficiency of Soybean under Phosphorus Stress. J. Plant Genet. Resour..

[21] Tarafdar J.C., Claassen N. (1988). Organic Phosphorus Compounds As a Phosphorus Source for Higher Plants Through the Activity of Phosphatases Produced by Plant Roots and Microorganisms. Biol. Fertil. Soils.

[22] Sanz-Luque E., Chamizo-Ampudia A., Llamas A., Galvan A., Fernandez E. (2015). Understanding nitrate assimilation and its regulation in microalgae. Front. Plant Sci..

[23] Karasov T.L., Chae E., Herman J.J., Bergelson J. (2017). Mechanisms to Mitigate the Trade-Off between Growth and Defense. Plant Cell.

[24] de Tombeur F., Pélissier R., Shihan A., Rahajaharilaza K., Fort F., Mahaut L., Lemoine T., Thorne S.J., Hartley S.E., Luquet D. (2023). Growth–defence trade-off in rice: Fast-growing and acquisitive genotypes have lower expression of genes involved in immunity. J. Exp. Bot..

[25] Wang D., Freschet G.T., McCormack M.L., Lambers H., Gu J. (2025). Nutrient resorption of leaves and roots coordinates with root nutrient-acquisition strategies in a temperate forest. New Phytol..

[26] Kim J., Kim J.H., Lyu J.I., Woo H.R., Lim P.O. (2018). New insights into the regulation of leaf senescence in Arabidopsis. J. Exp. Bot..

[27] Yadav V., Wang Z., Wei C., Amo A., Ahmed B., Yang X., Zhang X. (2020). Phenylpropanoid Pathway Engineering: An Emerging Approach towards Plant Defense. Pathogens.

[28] Gao Y., Dong X., Wang R., Hao F., Zhang H., Zhang Y., Lin G. (2024). Exogenous Calcium Alleviates Oxidative Stress Caused by Salt Stress in Peanut Seedling Roots by Regulating the Antioxidant Enzyme System and Flavonoid Biosynthesis. Antioxidants.

[29] Rao M.J., Zheng B. (2025). The Role of Polyphenols in Abiotic Stress Tolerance and Their Antioxidant Properties to Scavenge Reactive Oxygen Species and Free Radicals. Antioxidants.

[30] Tattini M., Guidi L., Morassi-Bonzi L., Pinelli P., Remorini D., Degl’Innocenti E., Giordano C., Massai R., Agati G. (2005). On the role of flavonoids in the integrated mechanisms of response of Ligustrum vulgare and Phillyrea latifolia to high solar radiation. New Phytol..

[31] Sato K., Nishikubo N., Mashino Y., Yoshitomi K., Zhou J., Kajita S., Katayama Y. (2009). Immunohistochemical localization of enzymes that catalyze the long sequential pathways of lignin biosynthesis during differentiation of secondary xylem tissues of hybrid aspen (*Populus sieboldii* × *Populus grandidentata*). Tree Physiol..

[32] Yao T., Feng K., Xie M., Barros J., Tschaplinski T.J., Tuskan G.A., Muchero W., Chen J.-G. (2021). Phylogenetic Occurrence of the Phenylpropanoid Pathway and Lignin Biosynthesis in Plants. Front. Plant Sci..

[33] Upchurch R.G. (2008). Fatty acid unsaturation, mobilization, and regulation in the response of plants to stress. Biotechnol. Lett..

[34] Hąc-Wydro K., Wydro P. (2007). The influence of fatty acids on model cholesterol/phospholipid membranes. Chem. Phys. Lipids.

[35] Santino A., Taurino M., De Domenico S., Bonsegna S., Poltronieri P., Pastor V., Flors V. (2013). Jasmonate signaling in plant development and defense response to multiple (a)biotic stresses. Plant Cell Rep..

[36] Jimenez-Aleman G.H., Almeida-Trapp M., Fernández-Barbero G., Gimenez-Ibanez S., Reichelt M., Vadassery J., Mithöfer A., Caballero J., Boland W., Solano R. (2019). Omega hydroxylated JA-Ile is an endogenous bioactive jasmonate that signals through the canonical jasmonate signaling pathway. Biochim. Et Biophys. Acta (BBA) Mol. Cell Biol. Lipids.

[37] Degenhardt B., Gimmler H. (2000). Cell Wall Adaptations to Multiple Environmental Stresses in Maize Roots. J. Exp. Bot..

[38] Cheng H., Jiang Z.Y., Liu Y., Ye Z.H., Wu M.L., Sun C.C., Sun F.L., Fei J., Wang Y.S. (2014). Metal (Pb, Zn and Cu) uptake and tolerance by mangroves in relation to root anatomy and lignification/suberization. Tree Physiol..

[39] Noctor G., Mhamdi A., Chaouch S., Han Y., Neukermans J., Marquez-Garcia B., Queval G., Foyer C.H. (2011). Glutathione in Plants: An Integrated Overview. Plant Cell Environ..

[40] Noctor G., Cohen M., Trémulot L., Châtel-Innocenti G., Van Breusegem F., Mhamdi A., Dietz K.-J. (2024). Glutathione: A key modulator of plant defence and metabolism through multiple mechanisms. J. Exp. Bot..

[41] de Bont L., Donnay N., Couturier J., Rouhier N. (2022). Redox regulation of enzymes involved in sulfate assimilation and in the synthesis of sulfur-containing amino acids and glutathione in plants. Front. Plant Sci..

[42] Chaudhary D., Agarwal H., Mishra A., Joshi N.C. (2024). Glutathione Homeostasis—A Prerequisite to Maintain Root System Architecture in Plants. J. Soil Sci. Plant Nutr..

[43] Rao F., Caflisch A. (2004). The Protein Folding Network. J. Mol. Biol..

[44] Lu Y.-L., Li Q.-Y., Xu X.-J., Zhu Y.-Y., Dong C.-X., Shen Q.-R. (2010). Responses of Tomato Seedling to Different Nitrogen Forms Supplied by Either Homogenous or Localized Culture. J. Nanjing Agric. Univ..

[45] Zhou H.M. (2019). Study on Drought Resistance of Dominant Shrubs and Mineral Elements of Dominant Shrubs in Arid Valley of the Minjiang River. Master’s Thesis.

[46] Li P.-F., Chen H., Huo J.-Z., Duan X.-C. (2005). Determination of Elements in Bitter Melon Leaves by ICP-AES. J. Tianjin Norm. Univ. (Nat. Sci. Ed.).

[47] Zhang Q., Zhou Z., Zhao W., Huang G., Liu G., Li X., Wu J. (2023). Effect of Slope Position on Leaf and Fine Root C, N and P Stoichiometry and Rhizosphere Soil Properties in Tectona Grandis Plantations. J. For. Res..

[48] Xiong Q.E. (2003). Plant Physiology Laboratory Manual.

[49] Li X.F., Zhang L.Z. (2016). Guidance of Plant Physiology Experiments.

